# Tennis Serve Constraints Delimit but Do Not Prohibit Individual Movement Strategies among Professional Players

**DOI:** 10.5114/jhk/203795

**Published:** 2025-09-23

**Authors:** Kaies Deghaies, Thibault Lussiana, Pierre Touzard, Loic Fourel, Simon Ozan, Cyril Brechbuhl, Cyrille Gindre, Benoit Bideau, Caroline Martin

**Affiliations:** 1 M2S Laboratory-Inria, University Rennes 2, Bruz, France.; 2 Research and Development Department, Volodalen, Chavéria, France.; 3 MPFRPV, Université de Franche-Comté, Besançon, France.; 4 Exercise Performance Health Innovation (EPHI), Université de Franche-Comté, Besançon, France.; 5 French Tennis Federation, Paris, France.; 6 Research and Development Department, Volodalen SwissSportLab, Aigle, Switzerland.

**Keywords:** tennis, sports performance, variability, motor control

## Abstract

In sport, copying the motion technique of top-level athletes, which is regarded as perfect and optimal, is a goal often targeted by coaches for their athletes. However, inter-individual variability has been shown to remain in sport motions even at the elite level, and its analysis is important to identify the different motor strategies used to respond to the same motor task. This study aimed to assess inter-individual variability of Center of Mass (CoM) vertical kinematics during the serve among a homogeneous population of twenty-two professional tennis players and to investigate its links with serve performance indicators. Vertical CoM trajectories, impact height and racket head velocity were analyzed using a marker-based motion capture system. Inter-individual variability was assessed using the coefficient of variation. The phase duration, the vertical CoM displacement and its maximal velocity showed greater variability during the loading phase compared to the acceleration phase. Strong correlations were found between vertical CoM parameters during the acceleration phase and ball impact height, but not during the loading phase. The serving task constraints seem to offer limited scope for inter-individual variability and tend to delimit CoM movement strategy during the acceleration phase. Conversely, individual strategies of CoM kinematics differ among professional players during the loading phase, which could be the result of the expression of organismic constraints. This inter-individual variability observed during the loading phase points towards a non-unique way of serving and the need for individualized coaching exercises.

## Introduction

For decades, coaches have followed the paradigm of the optimal kinematic movement pattern which promotes a unique and perfect technical movement to achieve high level sport performance. In this context, any inter-individual variability was considered a deviation from this optimal movement pattern and was consequently treated as undesirable (Brisson and Alain, 1996; Davids et al., 2003). However, recent investigations have shown that variability could instead be an attribute of very high-skilled athletes reflecting a high degree of flexibility and adaptability in the movement (Glazier and Lamb, 2017; Seifert et al., 2011). In sport biomechanics, these studies popularized the dynamical systems theory which stipulates that the way people act and interact with their environment is specifically influenced by the conditions and requirements of both the environment and the specific constraints of the task they perform (Newell, 1986). From then, inter-individual variability has been increasingly considered differently and individual’s actions are now positively viewed as emerging from the dynamic connections formed between individuals and their environment. Furthermore, Glazier and Mehdizadeh (2019) have highlighted the fact that, because most biomechanical collected data are based on means within groups, the inter-individual variability is frequently covered up and neglected, thus, leading to general probabilistic statements not applicable to specific athletes. When focusing on the general trends and patterns that emerge across the entire group, scientists may miss out on comprehending the full spectrum of movement possibilities within the group, potentially leading to incomplete or generalized conclusions (Glazier et al., 2006).

As a consequence, the analysis of movement variability has become a crucial area of research in sports biomechanics to disprove the idea of an optimal technique and to identify distinct coordination profiles (Bartlett et al., 2007). In professional athletes’ populations, the functional role of inter-individual variability was highlighted in diverse sport motions such as a back squat, breaststroke coordination, a golf swing and a table tennis forehand (Bańkosz and Winiarski, 2020; Glazier and Lamb, 2017; Kristiansen et al., 2019; Seifert et al., 2011), reflecting different individual movement strategies to maximize performance or reduce the risk of injuries while adapting to the task constraints. Even within the same sport movement, the level of inter-individual variability could also vary depending on the motion phases. In basketball shooting, the variability in shoulder kinematics is higher during the downward (loading) phase and lower during the upward (acceleration) phase (Ae, 2020). In the tennis serve, adult elite female players reduce movement variability in the lower limbs and the trunk between the ball zenith and the racket low point. But as they get close to impact, variability increases in the elbow which was interpreted as a sign of a compensatory function to ensure a suitable racket-ball contact (Whiteside et al., 2015). However, the role of inter-individual variability in performance has never been questioned in tennis and the optimal kinematic movement approach is still in vogue, especially regarding serve learning and training. Indeed, due to the closed-skill nature of the motion, as the golf swing can be (Glazier and Lamb, 2017), a better performance in a serve is seen through the prism of a better motion technique. Tennis biomechanists have undoubtedly contributed to the continuation of this approach by reporting mean values derived from different groups of players for lower and upper limb joints and/or segmental motions to describe the serve technique and understand their influence on serve performance indicators, i.e., ball speed and ball impact height (Fleisig et al., 2003; Martin et al., 2013; Whiteside et al., 2013). Consequently, a lot of coaches continue to convince players to change their technique to conform to their optimal picture of a good mechanical form (Anderson et al., 2021), referring to an internal model of the tennis serve rather based on pure technical knowledge than on understanding the functionality of biomechanics and how this operates for an individual player (Fetisova et al., 2021).

The serve is considered the most important stroke in tennis (Ma et al., 2013) as it initiates each rally and gives the server the opportunity to win free points and to dominate the opponent. It represents a complex motion that must respond to the constraints of a given task: “hitting a ball with a combination of high speed and accuracy to reach a horizontal target—the serve box, passing a vertical obstacle beforehand—the net”. The serve can be divided into two successive phases before impact: the loading and the acceleration (Kovacs and Ellenbecker, 2011). The loading phase of the serve initiates close to the ball toss, continues with the racket rise into a trophy position and ends with the storage of energy in the maximally flexed lower limbs. In the acceleration phase, leg extension transfers stored energy to the trunk and the dominant upper limb, increasing racket velocity and propelling the player towards the ball impact. During these two phases, the lower limbs play a crucial role, as they form the first link in the proximo-distal chain and provide the fundamental forces that drive the subsequent segments in the movement (Girard et al., 2005). It seems obvious that the lower limbs’ flexion-extension during these phases influence the vertical trajectory and velocity of the center of mass (CoM). CoM motion measurement can be of great interest in the description of sports movements such as the tennis serve because it essentializes a complex motion associated with multiple redundant degrees of freedom in the joint space. In jumping and/or overhead striking motions, analyzing CoM kinematics also provides a more comprehensive understanding of the whole body's dynamics in relation to performance. For example, analysis of CoM kinematics in countermovement jumps and volleyball spikes has shown that the CoM acceleration produced by leg action is a crucial factor in jump height (Kipp and Kim, 2020; Zahálka et al., 2017). Regarding the tennis serve, the CoM vertical velocity at the instant of the maximal vertical power during the acceleration phase of the leg drive is significantly and moderately correlated with ball impact height (Fourel et al., 2024).

This study aimed to evaluate inter-individual variability in vertical positions, ranges of motion, maximal CoM velocity and duration of the loading and acceleration phases of the tennis serve and investigate the links between this variability and performance indicators (maximal racket head velocity and ball impact height). It was expected that the duration and the vertical CoM variables of the loading phase would be more variable than those of the acceleration phase and would not correlate with serve performance indicators. We hypothesized that the loading phase would give athletes more freedom to express individual CoM motor kinematics than the acceleration phase, without affecting serve performance.

## Methods

### Participants

Power analyses indicated that the minimum sample size to yield a statistical power of at least 0.80 with an alpha of 0.05 and a correlation coefficient of r = 0.75 was 11 for a Pearson correlation and 14 for a Spearman correlation. Here, a total of 22 male professional tennis players (19 right-handed and 3 left-handed; age: 22.8 ± 3.5 yr; body height: 1.89 ± 0.08 m; body mass: 80.2 ± 7.8 kg) with a mean ATP (Association of Tennis Professionals) ranking of 231 (highest 17; lowest 565) voluntarily took part in the study, ensuring that our population was large enough to test correlations. All players served using a foot-up technique in which they brought the back foot up to meet the front foot during the ball toss, prior pushing against the floor. Participants were free from any injuries or pain that could potentially affect their serve performance. Before the experiment, all participants provided informed consent. The study was approved by a local ethics committee (Comité de Protection des Personnes Ile de France I, Paris, France) (approval code: 2021- A02133-38; approval date: 15 December 2021) and conducted under the 1975 Declaration of Helsinki.

### Design and Procedures

Following a warm-up session, each player was fitted with 38 retro-reflective body markers (14 mm in diameter), placed on specific anatomical landmarks based on previously published data (De Leva, 1996). These markers were used to determine joint centers and the CoM for selected body segments. Five additional markers were positioned on the racket (Martin et al., 2014). Additional flat reflective markers were located on the ball to obtain ball impact instant. After a few trial serves, each player performed five successful flat serves in a target area (1 m x 2 m bordering the “T” zone) in a deuce service box for right-handed players or in an ad service box for left-handed players. Participants used their own racket during motion capture. Three-dimensional marker trajectories were recorded at 300 Hz using a 23-camera Qualisys motion analysis system (Oqus 7+, Qualisys AB, Gothenburg, Sweden). The baseline center was the global origin: the positive X-axis extended right, the positive Y-axis pointed toward the net, and the positive Z-axis pointed upward.

### Measures

**Figure 1:**
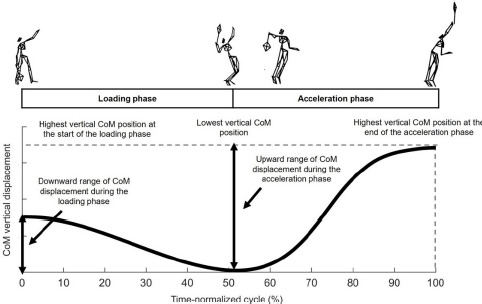
Illustrations of key instants, phases of the serve, and selected CoM variables. *Notes: CoM: Center of Mass*

After the capture, three dimensional coordinates of the markers were reconstructed with Qualisys Track Manager software with a residual error less than 1 mm. Data were then exported to Matlab (Version R2018a, Mathworks, Natick, Massachusetts, USA) for processing. The 3D coordinate data of the body and racket markers were smoothed using a low pass filter with a cut-off frequency of 20 Hz. The serve was divided into the loading and acceleration phases. The follow-through phase was not considered in our analysis because it is not an active phase of the serve motion. The loading phase was defined from the initial local maximum in the vertical CoM position (near ball release instant) to the lowest vertical CoM position at a fully loaded lower body position (near trophy position instant). The acceleration phase was defined from the end of the loading phase until the highest vertical CoM position (near impact instant) (Figure 1). Data points were interpolated before averaging and time was normalized from the start of the loading phase to the end of the acceleration phase.

Eleven dependent kinematic variables were selected to analyze the inter-individual variability throughout the serve. Ball impact height, vertical CoM positions at various key-instants (start and end of the loading and acceleration phases), downward and upward ranges of CoM displacement during the loading and acceleration phases (Figure 1) were normalized by player’s body height. We focused our analysis on the vertical CoM variables to study inter-individual variability, as the definition of serve phases (loading and acceleration) is mainly defined by the vertical action of the player’s body (Bahamonde and Knudson, 2001). All variables were extracted from each serve and later averaged across the five successful trials of each player.

### Statistical Analysis

Mean and standard deviation (SD) were computed for all variables. The Shapiro-Wilk test was used to assess the normality of the data distribution. Inter-individual variability was evaluated using the coefficient of variation (CV) calculated as the ratio of the standard deviation to the mean and was expressed as a percentage. Based on the thresholds set by Bańkosz and Winiarski (2020), a CV below 20% indicated low variability, a CV between 20% and 40% indicated medium variability, a CV between 40% and 60% indicated high variability and a CV higher than 60% indicated very high variability. When data did not follow a normal distribution and followed a log-normal distribution, CV was calculated as follows:


$$CV=\sqrt{e^{s}-1},$$

where *s* was the variance of the natural logarithm (ln) of the data. To verify that the variability observed among players was predominantly due to inter-individual differences and was only minimally influenced by individual variability resulting from the five successful serves, we also measured the intra-individual variability by calculating the mean of the CV for each player across the five performed serves.

Pearson’s correlation coefficients were used to assess relationships between CoM variables and serve performance indicators (maximal racket head velocity, ball impact height). When the normality assumption failed, Spearman’s correlation coefficients were used. Based on commonly used thresholds, correlation was considered weak, moderate, and strong, when the coefficient was between 0.25 and 0.5, 0.5 and 0.75, and above 0.75, respectively (Portney and Watkins, 2009). Statistical analyses were performed using Statistica software (Statistica 12, Statsoft Inc., Tulsa, USA). The level of significance was set at *p* = 0.05 and adjusted to *p* = 0.0028 (0.05/18) using a Bonferroni correction to control type I error.

## Results

### Maximal Racket Head Velocity and Ball Impact Height

The inter-individual variability concerning maximal racket head velocity and ball impact height was low (CV: 5.3% and 1.8%, respectively) (Table 1) ensuring performance homogeneity in our population.

**Table 1:** Descriptive data of maximal racket head velocity, ball-impact height, and CoM kinematic parameters.

	Mean	SD	CV (%)	CV intra† (%)
***Serve performance indicators***				
Maximal racket head velocity (m•s^−1^)	47.5	2.5	5.3	1.4
Ball-impact height (% of BH)	147.6	2.6	1.8	0.7
***Vertical CoM positions***				
Highest vertical CoM position at the start of the loading phase (% of BH)	56.1	2.2	4.0	0.4
Lowest vertical CoM position at the start of the acceleration phase (% of BH)	50.6	1.9	3.8	0.8
Highest vertical CoM position at the end of the acceleration phase (% of BH)	66.3	2.1	3.2	1.0
***Vertical CoM displacements***				
Downward range of CoM during the loading phase (% of BH)	–5.5	2.3	41.6	8.6
Upward range of CoM during the acceleration phase (% of BH)	15.8	3.4	21.3	5.7
***Maximal vertical CoM velocities***				
Maximal downward velocity of CoM during the loading phase (m•s^−1^)	–0.3	0.1	42.2	10.3
Maximal upward velocity of CoM during the acceleration phase (m•s^−1^)	1.7	0.2	14.6	3.3
***Duration***				
Loading phase duration (s)	0.56	0.12	20.7	5.4
Acceleration phase duration (s)	0.40	0.07	18.5	4.9

† Coefficients of variation calculated for intra-individual variability from five successful serves. Notes: SD: standard deviation; CV: coefficient of variation; CV intra: intra-individual coefficients of variation; BH: body height; CoM: Center of Mass

### Vertical Position and Displacement of CoM During Loading and Acceleration Phases

**Figure 2:**
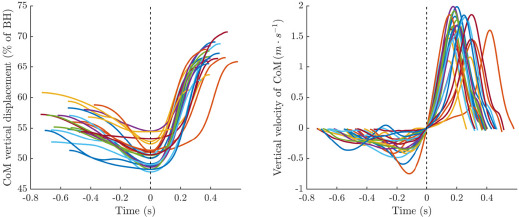
Normalized vertical CoM displacement (left) and velocities (right) for each player. Time is shown in absolute values, starting from the lowest CoM position (dotted line), where velocity is zero. *Notes: CoM: Center of Mass, BH: body height*

The inter-individual variability concerning CoM vertical positions at the start and the end of the loading phase and at the end of the acceleration phase was low (CV: 4.0%, 3.8% and 3.2%, respectively). The CV of the downward range of CoM during the loading phase was high (41.6%) while it was medium (21.3%) for the upward range of CoM during the acceleration phase. The individual trajectories of the CoM vertical position during each phase of the serve are illustrated in Figure 2.

### Maximal CoM Velocities During Loading and Acceleration Phases

The CV of the maximal downward velocity of the CoM during the loading phase was superior to that of the maximal upward velocity during the acceleration one (42.2% *vs*. 14.6% respectively). Figure 2 shows the evolution of CoM vertical velocity during each phase of the serve for each player.

### Duration of Loading and Acceleration Phases

The inter-individual variability in the phase duration was higher in the loading than in the acceleration phase (20.7% *vs*. 18.5%, respectively). Figure 2 shows the absolute duration of each phase for each player.

### Relationships between Serve Performance Indicators and CoM Variables

There was no significant correlation between CoM variables during the loading phase and serve performance indicators. Conversely, there were four significant correlations between CoM variables during the acceleration phase and ball impact height. Indeed, ball impact height showed a negative correlation with the lowest vertical CoM position at the start of the acceleration phase, a positive correlation with the highest vertical CoM position at the end of the acceleration phase, a positive correlation with the upward range of CoM displacement during the acceleration phase, and a positive correlation with the maximal upward velocity of CoM during the same phase (*r* = −0.65, *r* = 0.78, *r* = 0.87, *r* = 0.79, respectively) (Table 2). No CoM variable in the acceleration phase was correlated with the maximal racket head velocity.

**Table 2:** Pearson's and Spearman’s coefficients of correlation between CoM variables and serve performance indicators

	Maximal racket head velocity (m•s^−1^)	Ball-impact height (% of BH)
***Vertical CoM positions***		
Highest vertical CoM position at the start of the loading phase (% of BH)	−0.49	–0.37
Lowest vertical CoM position at the start of the acceleration phase (% of BH)	–0.11	–0.65***
Highest vertical CoM position at the end of the acceleration phase (% of BH)	–0.17	0.78***
***Vertical CoM displacements***		
Downward range of CoM during the loading phase (% of BH)	–0.31	0.20
Upward range of CoM during the acceleration phase (% of BH)	–0.01	0.87***
***Maximal vertical CoM velocities***		
Maximal downward velocity of CoM during the loading phase (m•s^–1^)	0.19	–0.10
Maximal upward velocity of CoM during the acceleration phase (m•s^–1^)	0.08	0.79***
***Phase duration***		
Loading phase duration (s)	0.01	0.07
Acceleration phase duration (s)	–0.13	0.16

Notes: CoM: Center of Mass. *** p < 0.001

## Discussion

This study aimed to evaluate inter-individual variability in CoM vertical kinematics for the loading and acceleration phases of the tennis serve and explore its relationships with serve performance indicators (maximal racket head velocity and ball impact height) in professional players. The inter-individual variability of CoM vertical displacements, velocities and phase duration was higher during the loading than the acceleration phase. Additionally, no correlation was found during the loading phase between CoM variables and impact height nor maximal racket velocity, reflecting different motor strategies unrelated to serve performance.

The low CV observed for mean maximal racket head velocity (5.3%) highlights a homogeneous performance level within the group. The mean maximal racket head velocity in our study was in line with previous findings for professional players (Reid et al., 2011). Moreover, ball impact height showed very low inter-individual variability (CV: 1.8%), confirming that it is one of the most stable criteria among elite players. This stability is primordial for performance, as hitting the ball higher provides a greater margin over the net, improving serve effectiveness (Vaverka and Cernosek, 2013). The mean ball impact height in our study (147.6 ± 2.6% of BH) closely matched 149 ± 5.6% of BH reported for elite players (Reid et al., 2011).

The greater inter-individual variability observed during the loading phase in CoM vertical organization shows that the beginning of the serve motion is individual-dependent. Such variability has no impact on serve performance since CoM vertical kinematics do not significantly correlate with impact height nor maximal racket velocity during this phase. Intra-individual variability remains low during the loading phase and is not the cause for individual differences. Our results rather show that the inter-individual variability during the loading phase supports the idea that there is not a single ideal serve to be imitated. Instead of employing the ‘one size fits all’ approach, tennis coaches should accept a certain amount of movement variability in this phase. It seems more interesting to allow players to explore possible solutions, particularly in the early stages of learning, rather than having the coach impose a unique model on them. Drills introducing variability in the loading phase could for example be developed by coaches to help players find their own path. Since our results show a significant negative correlation between the vertical CoM position at the end of the loading phase (beginning of the acceleration phase) and the impact height, the loading phase can be summed up as follows: no matter how the player does it, the aim is to get the CoM sufficiently low before pushing of the legs. Future studies must continue to identify the elements linked to serve performance (and therefore the elements considered as technical errors) between those reflecting idiosyncrasies.

During the acceleration phase, CoM vertical kinematics correlate with impact height while being less variable. This phase is therefore crucial for serve performance and leaves less room for individual expression and motor strategies. It is likely because the action of pushing upwards with maximum velocity to reach the highest CoM position is directly related to the serving task's requirements (the higher the impact height, the greater the margin over the net). Our results are in agreement with the low variability during the acceleration phase previously observed by Whiteside et al. (2015) for the lower limbs in female tennis players. According to Seifert et al. (2013), the critical phases of motion can limit individual differences, and force athletes to adapt their personal traits to meet specific task requirements and environmental constraints. Even if the acceleration phase constraints all players to adopt a similar strategy, our results demonstrate that a slight increase in upward maximal CoM velocity enables to gain a few extra centimeters in ball impact height, which is crucial for improving net clearance and the serve percentage (Vaverka and Cernosek, 2013). Since CoM kinematics in the serve motion are intimately linked to the leg drive, our findings also suggest the importance of a rapid leg extension in enhancing serve impact height (Girard et al., 2007). Fourel et al. (2024) also highlighted the importance of the relative net vertical impulse and the CoM vertical velocity at the instant of maximal vertical power since they are good predictors of serve impact height. Finally, none of the CoM variables measured during the acceleration phase correlated with maximal racket head velocity. This supports the idea that the vertical dimension of the serve motion is not preponderant for improving the power of the stroke (Fourel et al., 2024).

**Figure 3:**
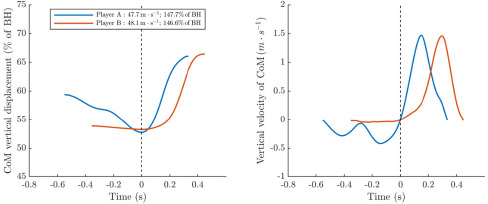
Normalized vertical CoM displacement (left) and vertical CoM velocity (right) during the serves of Player A and Player B, both with similar maximal racket head velocity and ball impact height. Time is shown in absolute values, starting from the lowest CoM position (dotted line), where velocity is zero. *Notes: CoM: Center of Mass, BH: body height*

The dynamical systems theory, which stipulates that variability is a mechanism allowing players to adapt their movements as a function of organismic, environmental and task constraints (Newell, 1986), provides a framework of interpretation for our data. With regard to the tennis serve, the task and environmental constraints were identical for all players in our study. They included the traditional dimensions of the court and net height (0.914 m), specific instructions (1^st^ serves must bounce inside the T zone of the service box with speed and accuracy) and the environment where the experiment took place (indoor tennis court with artificial light, no opponent, no wind). Organismic constraints lead to differences between players due to anthropometric variables, body composition, muscle architecture, specific physical characteristics (maximal strength, muscle tendon properties, neuromuscular power), and motor preferences or preferential contraction patterns. The impact of physical and anthropometric characteristics (body height, body mass, arm span) on serve performance has already been analyzed (Fett et al., 2020), whereas the influence of preferential motor or contraction patterns has never been investigated in tennis. Yet, the analysis of motor preferences is growing in sports research (Hanley et al., 2022; Patoz et al., 2020). For instance, it has been shown that stretch-shortening cycle characteristics in running and the ability to effectively store and release energy are different between two motor profiles referred to as the "aerial or plyometric profile" and the "terrestrial or concentric profile" (Lussiana et al., 2017). Our results suggest the presence of subtle individual signatures (Kelso, 1995) especially when we isolate the cases of two professional players that might reflect distinct motor profiles or contraction patterns (Figure 3). They display identical serve performance, i.e., identical maximal racket head velocities and ball impact heights (only 1.1% BH difference). Additionally, they achieve similar CoM rise ranges (13.3% *vs*. 13.2% of BH) and reach about the same upward maximal CoM velocity (1.49 *vs*. 1.47 m⋅s^−1^). However, player A's movement strategy tended towards a plyometric mode of contraction (spring like behavior, “V-shaped” curve), in contrast to player B who demonstrated a more concentric mode of contraction (“S-shaped” curve). Moreover, player A had a greater CoM drop range (−6.6% of BH) compared to Player B (–0.6% of BH), and a downward maximal CoM velocity more than four times faster (–0.43 *vs*. –0.10 m⋅s^–1^). Since serve performance was similar between these two players, these results illustrate the concept of equifinality in movement which states that different motor coordination could lead to the same outcome (Müller and Loosch, 1999; Bańkosz and Winiarski, 2020). From a coaching point of view, the implication of the above findings includes the necessity of individualized training and physical preparation programs. The training exercises performed by each professional player should be done in a way that replicates their individual movement patterns, to ensure movement specificity. Since the height of the ball toss delimits the time available for the loading phase, our results encourage coaches to be cautious about this element in relation to the preferential contraction pattern of their player.

Considering the limitations of our research, it is worth acknowledging that we did not measure the physical capacities of each player that could also provide more comprehensive insights into inter-individual variabilities, particularly regarding the abilities related to the storage and release of elastic energy. Intra- and inter-individual variability between different levels of expertise and more complex kinematic variables (for example 3D joint angles and angular velocities) should also be investigated in the future. Clustering-based methods including other biomechanical variables could be relevant to see whether individuals could be distributed into distinct groups of motor coordination. Finally, the present study was focused on inter-individual variability among players serving with a foot-up technique. Since professional players can also serve with their feet fixed on the ground until the take-off (called the foot-back technique), the inter-individual variability associated with this other stance technique should also be analyzed to see whether one type of a stance is better suited to one preferential contraction pattern.

## Conclusions

In our study, we found greater variability in vertical CoM displacements and velocities during the loading compared to the acceleration phase, suggesting that individual signature patterns emerged during the loading phase of the tennis serve. Our results support the idea that the constraints of the serving task faced by the tennis player tend to delimit movement strategy during the acceleration phase. These results emphasize the need for individual analyses to better understand motor strategies used during the tennis serve and their biomechanical impact on performance. From a practical point of view, our results encourage coaches to work with their players in a tailored-made fashion to optimize their motor strategies when serving, taking into account both the constraints of the task and the specific individual characteristics of each player.
